# Rabies in rural northeast India: A case report emphasising the urgency of the One Health approach

**DOI:** 10.1016/j.onehlt.2024.100850

**Published:** 2024-07-03

**Authors:** Parimala Mohanty, Prasanta Kumar Boro, Samira Heydtmann, Salome Durr, Harish Kumar Tiwari

**Affiliations:** a Jyoti and Bhupat Mehta School of Health Science and Technology, Indian Institute of Technology Guwahati, Guwahati, Assam, India; b Department of Veterinary Medicine, Lakhimpur College of Veterinary Science, Joyhing, North Lakhimpur, Assam, India; c Vetsuisse Faculty, University of Zurich, Switzerland; d Veterinary Public Health Institute, University of Bern, Switzerland; e Sydney Medical School, Faculty of Medicine and Health, University of Sydney, NSW, Australia; f DBT-Wellcome Trust India Alliance Intermediate Fellow, Hyderabad, Telangana, India

**Keywords:** Rabies, Dog bite, Post-exposure prophylaxis, Healthcare infrastructure, Rabies death, Case report

## Abstract

Dog-mediated rabies is endemic in India. The country records the highest mortality due to dog-bite-related rabies despite the availability of interventions to prevent deaths. We present a case study of the death of a 59-year-old man in a suburban town of Northeast India after a dog bite from an owned pup. Through this case study, we investigate various omissions and commissions in communities and health professionals that make rabies rampant in India. The circumstances surrounding the death were investigated by interviewing the wife, relatives, neighbour, the hospital/nursing home where the bite case was reported, the district Rapid Response Team (RRT), and the Veterinary and Animal Health Department Officer and through the information recorded in the disease outbreak report. While the biting animal was not vaccinated and had no restriction over its movement imposed by the owners, the response of the hospital staff and public authorities was delayed and inadequate. A poignant reminder of the complexities surrounding dog-mediated rabies in India, this case study calls for a holistic protocol to address dog bites through ensuring the One Health approach encompassing education, provision of post-exposure prophylaxis (PEP) and canine rabies vaccines for dogs, promotion of responsible dog ownership, and intersectoral collaboration. Moreover, strengthening communication channels through effective data exchange and encouraging synergy among healthcare, veterinary, and public health sectors is indispensable to maximize the impact of rabies prevention and control interventions.

## Highlights

• Northeast India is a hotspot for dog-mediated rabies.
• Case study reveals gaps in wound management and prevention.
• Lack of awareness and responsible dog ownership contribute to fatalities.
• Novel insights on rabies dynamics in underserved regions.
• Urgent need for One Health approach through intersectoral collaboration and communication to control dog-mediated rabies.

## 1 Introduction

Rabies causes mortality of more than 59,000 people globally every year, one-third of which are from India [1,2]. The disease is preventable through post-exposure prophylaxis (PEP) [3,4], and the risk of rabies is mitigated by adequate toileting of the dog bite wound [5,6]. India has one of the largest populations of free-roaming dogs (FRD) in the world [7,8], including semi-owned and owned dogs with no restriction over their movement, making it a high-risk country for dog-mediated rabies.

Lack of awareness about the disease among communities, inadequate dog-bite wound management practices by paramedical staff, and the ubiquitous presence of FRD contribute to high mortality in India from rabies [9]. Despite remarkable improvements in the accessibility and affordability of PEP, human deaths continue due to poor disease awareness, especially among the socio-economically weaker population [10,11]. Therefore, dog-mediated rabies presents a persistent threat that demands immediate attention from the affected communities and health professionals [1,12].

Few case studies provide local insights or highlight the gaps leading to human mortalities due to dog bites in northeast India [13]. Such scarcity of published local cases stymies learning the complex dynamics associated with dog-bite-related fatalities in India. Here, we present a case study from northeast India where an individual succumbed to rabies after being bitten by an adopted pup. The study highlights the neglect at the intersections of human and animal health sectors, gaps in prevention, slow response mechanisms, and awareness deficit about rabies and responsible dog ownership prevailing in the region.

## 2 Case presentation

**Fig. 1:**
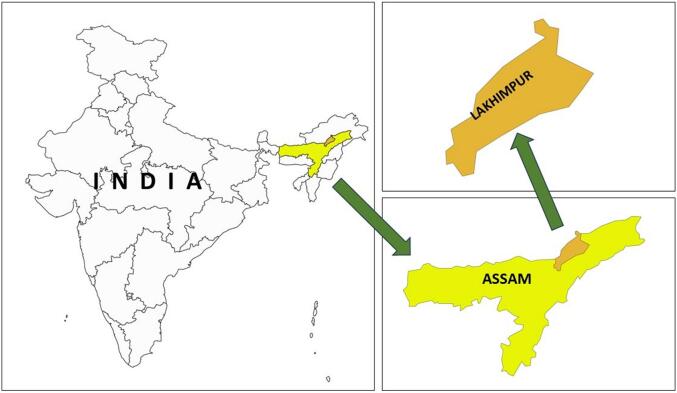
Map of India indicating Assam state of northeast India illustrating the location of Lakhimpur district where the death of an individual following a bite from an owned pup took place.

This case study is from Lakhimpur district (26°- 27° N, 93°-94° E), Assam, India. It includes incidences from 23 May to 13 June 2023 (Fig. 1). The case study includes information collected through the disease outbreak report and open discussions with the victim's relatives, neighbours, the village head, the hospital, the nursing home staff, the district Rapid Response Team (RRT), and the Veterinary officer of the local veterinary dispensary. The CARE guidelines checklist is provided as a supplementary file.

### 2.1 Patient and dog information

A 59-year-old man frequently visited the construction site to monitor the work progress on the house he was building adjacent to the bypass road in the outskirts of North Lakhimpur district of Assam, India, where he was bitten by the neighbour's pup on 23 May 2023.

The pup's owner had education up to matriculation level (secondary school), but his wife was illiterate. The two children in the family attended a nearby primary school. The owner frequently travelled out of the town for work, and the family adopted a local pup to protect them from approaching outsiders and wild animals by sounding alerts. The pup was born to an owned bitch with no veterinary or vaccination records, with restricted movement to the owner's household from birth to adoption. However, after the adoption, the pup was allowed to roam freely and return to the new owners to feed on their leftovers. The family that adopted the pup had limited knowledge about responsible dog ownership and opted not to immunise the pup against rabies. They later cited financial constraints as the reason for not immunising the pup.

### 2.2 Dog-bite and clinical course

On 23 May 2023, the pup attacked the victim unprovoked, causing deep bite wounds on the forehead, eyelid, leg, and groin. The pup was around 5–6 months during the incident. The patient reported to the Lakhimpur Medical College and Hospital (LMCH), where he refused hospitalisation, citing unsatisfactory care by the staff. The victim preferred a private nursing home where paramedical staff conducted antiseptic dressing and closed the bite wound with sutures. The patient was administered rabies immunoglobulins (RTG), PEP, and parenteral antibiotics at the nursing home and discharged on 26 May 2023 as his condition reportedly improved.

The biting animal (pup) showed progressive clinical signs of rabies with increased aggressive behaviour, excessive drooling, and disinclination to eat or drink. The pup died two days later (25 May 2023). Although the remaining family members did not report bites, exposure through contact is plausible.

### 2.3 Public health response

The delayed notification of the death of the pup to the medical Rapid Response Team (RRT) (comprising the Disease Surveillance Officer, Epidemiologist, and laboratory technicians, a standing team to address disease outbreaks in the area) and the veterinary department resulted in the animal buried without collecting the brain samples. Hence, the cause of death of the pup remained undiagnosed. The RRT advised the bitten individual and the family members to complete the PEP course. The sutures, however, were not removed, and the wound was left to heal with the previous dressing.

### 2.4 Clinical signs and symptoms

**Fig. 2:**
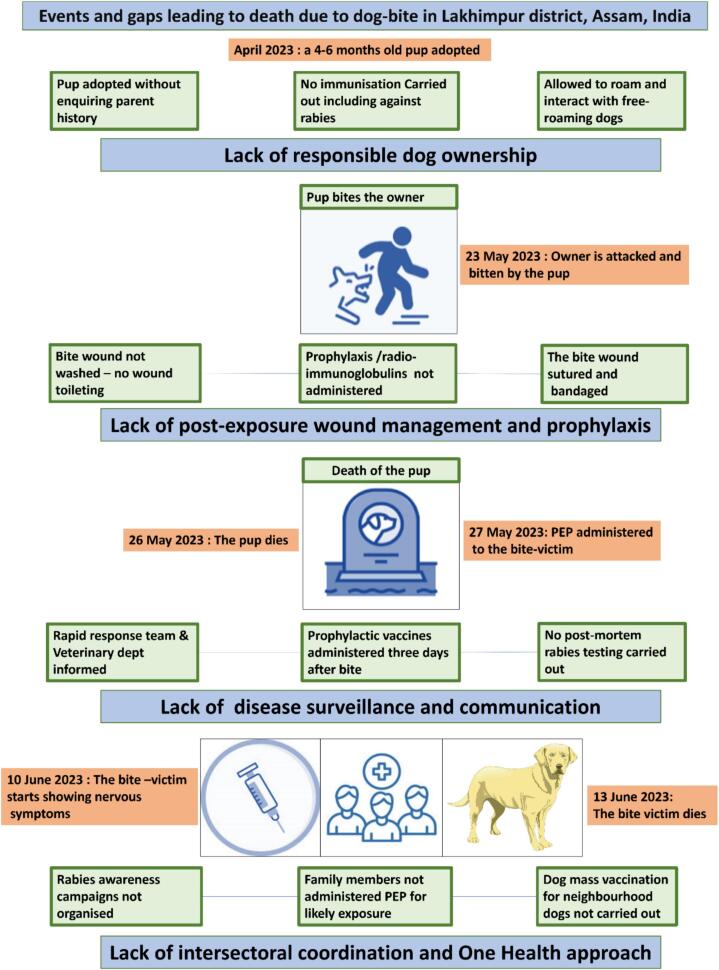
Infographics of events and gap leading to death due to bite injuries from an owned pup that took place in Lakhimpur district of Assam State, India in May–June 2023.

Despite administering RIG and four doses of PEP, the patient complained of headache, high fever, hoarse voice, drooling saliva, and difficulty in drinking or swallowing on 12 June 2023. Gradually, his behaviour became violent as days passed, and he died on 13 June 2023. Table 1 summarises the sequence of events of the case study, and the associated infographics highlighting the gap leading to death due to a dog bite in the Lakhimpur district of Assam State, India, is depicted in Fig. 2.

**Table 1:** The sequence of events and the action taken during an incident of death due to a dog bite in the Lakhimpur District of Assam State, India during May–June 2023.

Date	Description of the event	Action taken
23-May-23	Neighbour's pup bites the victim leaving deep wounds on leg and face.	The patient was not satisfied with the treatment he received at the Government hospital, so the individual goes to a local private clinic where antiseptic dressing of wound, parenteral antibiotics, and RIG, PEP administered.
25-May-23	The biting animal dies after showing enhanced clinical signs of rabies such as progressive aggressive behaviour, disinclination to eat, and excessive drooling of saliva.	The dead pup is buried without testing for rabies. The Veterinary department and Rapid Response Team (RRT) are informed very late regarding the death of the pup potentially from rabies.
26-May-23	The victim is discharged after 2 doses of ARV and improved health condition.	RIG and PEP is administered to the individual; wound is left to heal with sutures and bandage in place.
30-May-23 & 6-Jun-23	3rd and 4th doses of ARV given in the private hospital.	Follow-up PEP administered without assessment of patients over all condition.
10-Jun-23	The bitten individual complains of severe headache, and high fever	Symptomatic treatment was reportedly provided at home.
12-Jun-23	The patient feels difficulty in swallowing and drinking, the voice goes hoarse, and shows irritable and violent behaviour.	The patient is taken to a local medical specialist clinic. He was advised for CT scan in LMCH.
13-Jun-23	He was bought to LMCH for CT scan where he died in the morning 10.30 am.	Death due to rabies from dog-bite is reported to the district medical authorities and an event alert was generated in the Integrated Health Information Platform (IHIP) portal at LMCH

## 3 Discussion

This case study highlights the cardinal commissions and omissions prevalent in countries such as India, which are endemic to rabies. Moreover, with few case studies available in the region, dog-mediated rabies remains a neglected disease as several cases go unreported in northeast India [[14], [15], [16]]. This case report identifies inadequate post-bite wound management practices, driven by a lack of awareness and protocols, which result in a fatal outcome. The absence of responsible dog ownership, low vaccination coverage of owned dogs, and failure to conduct diagnostics for likely rabies death points to gaps in arresting the disease in dogs. A comprehensive One Health approach that promotes responsible dog ownership, including vaccination, wound treatment, and PEP administration, and simultaneously fosters intersectoral communication and collaboration needs implementation to prevent such incidences [17,18].

In the order of significance of the actions that led to rabies death, the lack of seriousness on the part of the hospital staff to attend to dog bite injury, prompting the patient to approach a local private nursing home, ranks foremost. Immediate wound toileting followed by PEP could have prevented the patient's death. The non-adherence to wound toileting could be attributable to a lack of training for the paramedical staff regarding the seriousness of dog bites. Paramedical staff are initially taught lessons during their induction into the profession. However, lack of refresher training leads to poor reinforcement of essential practices such as wound washing, especially for animal-bite cases. The slow response of the paramedical staff could be due to resource constraints in government sector hospitals, such as staff shortage, availability of specialists, and overburdened patient flow [[19], [20], [21]]. The private nursing home staff failed to comprehend that washing with soap water is mandatory for a dog-bite wound, and suturing of the wound is contraindicated [6,22]. A study by Tiwari et al. 2018 highlighted the necessity of incorporating dog-bite wound management into paramedical staff's educational curriculum, alongside routine refresher training. The latter is essential to maintain optimal nursing practices for the paramedical staff. Studies from rural areas have advocated that knowledge of wound management practices among paramedical staff needs to be updated [23,24]. We recommend refresher training to the paramedical staff on wound severity, classification, necessity of wound toileting, and proper administration of PEP with the Anti-rabies vaccine (ARV) vaccine, Rabies immune globulin (RIG) for effective management of dog-bite wounds [[25], [26], [27]]. Although it appears that the availability of ARV was not a concern, other mandatory requirements ensuring quality immunisation, such as not maintaining a cold chain, could have been a factor in reducing the efficacy of PEP administered within 48 h of the bite. The influences that diminish the vaccine quality due to poor storage and transportation need investigation, especially in remote and rural areas [28].

The availability of RIG or PEP is not a cause of concern in this study, as the patient did receive prophylactic administrations. The improved policies instituted during the 12th Five-year plan of the Government of India [29], have facilitated PEP availability even in the remotest parts of India [[30], [31], [32]]. The untimely PEP and RIG administration for category III wounds indicates poor dog-bite wound management practices rather than the former's unavailability [33,34]. Hence, a lack of knowledge about the disease and inadequate post-bite practices need attention to avoid fatalities due to dog-bite-related rabies [31,35,36].

Further, this case study highlights the need for coordination and communication between relevant stakeholders. One would expect the pup's death to trigger action from the RRT and the veterinary department. However, a lack of information flow between the human and animal health sectors likely delayed such response. Failure to collect brain samples to confirm the pup's death due to rabies is a gap in effective surveillance of dog bites resulting in rabies [[37], [38], [39]]. Without a proper diagnosis of the pup's death, crucial information on potential transmission to the family remains dubious and incomplete [[40], [41], [42], [43]] - such inaction limits to alert individuals inadvertently at risk of rabies infection.

Further, the likelihood of the pup biting other animals, including livestock, cannot be ruled out. A recent study showed that cattle are one of the most affected livestock by dog-mediated rabies in Assam [44,45]. Furthermore, poor surveillance efforts enable the spread of the disease in both animal and human populations [46,47]. Collaboration and communication between veterinarians and health authorities can ensure robust surveillance and timely response [[48], [49], [50], [51]]. The RRT and veterinary department officials admitted that efforts towards rabies awareness and dog mass vaccination programs in the district are yet to target free-roaming dogs, particularly in rural areas. Such interventions could have increased vaccination coverage by including dog owners who ignored their pets' immunisation due to cost constraints.

A significant omission on the part of the pup owner is failing to inquire about the immunisation history of the pup. Non-adherence to vaccination, even after adoption, indicates the casual attitudes of the owner that not only undermine the pup's welfare but pose a risk to the former's safety. In this instance, not vaccinating the dam and the pup was attributed to the unaffordability of vaccine cost. Monetary constraints are often cited as a barrier to canine rabies vaccination, as reported in rural Baramati, Maharashtra, India, where only 12% of owned dogs were vaccinated [52] and African countries endemic to dog-mediated rabies [53,54].

Further, in India, the tenets of responsible dog ownership are seldom adhered to and rarely accorded any importance, especially for a non-pedigree adopted pup. Developing and widely circulating the dog adoption protocol to guide prospective dog owners may help increase awareness about pet adoption. A lack of comprehensive information about the history of the adopted dogs stymies the ability to assess dogs' likely behaviour. A study from Punjab, India, reveals regional disparities in the adoption process, with variations in the adoption practices of urban and rural dog owners [55], suggesting that not all regions have standardised protocols or comprehensive information-sharing mechanisms regarding dog adoption. Despite the challenges associated with unknown histories, FRDs can adjust to domestic life and become excellent companions [56], but pets with restricted movement present the risk of disease transmission [57]. A pet with limited or no restriction over movement has higher odds of interaction with an infected FRD, resulting in potential transmission to the owners [58]. In the present case, the pup roamed without restriction and freely interacted with other FRDs, potentially exposing it to infections while adversely affecting dog-owner bonding [[59], [60], [61]]. Other studies have reported an increased likelihood of bites with unrestricted dog movement [12,62,63]. Admittedly, a lack of leash laws and enforcement mechanisms is a significant concern in countries with huge FRD populations [64].

On the contrary, local motivations and attitudes of dog owners in rural areas are crucial when considering regulations on confining dog movement on a leash [59,65]. While the owners' option to keep their dogs unrestricted, especially in rural areas, cannot be stopped owing to their utility as guarding dogs, there are chances of potential dog-wildlife conflict [65]. A tailored, culturally sensitive, responsible dog ownership campaign inclusive of local insights and regulatory measures would ensure public safety in the areas near wildlife where chances of spreading the rabies virus from wildlife to domestic animals are high [[66], [67], [68]].

The One Health approach is essential to address the challenge of rabies effectively. Such an approach involves implementing actions in parallel, such as promoting responsible dog ownership, dog vaccination, wound treatment, and PEP administration, and fostering coordination between different sectors. Additionally, streamlining communication channels between veterinary and human healthcare sectors facilitates the timely sharing of information, enabling more effective management of cases involving potential rabies exposure. While addressing responsible dog ownership and vaccination is crucial, avoiding redundancy and streamlining messaging to ensure clarity and focus in initiatives would ensure that enhanced efforts and efficient allocation of resources address issues such as inadequate post-bite wound management and rabies prevention.

## 4 Conclusion

This case report contributes to understanding the rampant lack of knowledge about rabies and inadequate practices of dog-bite wound management. In northeast India, where studies on rabies are conspicuously absent, this study highlights the urgency of initiating measures to enhance knowledge among communities and paramedical staff to prevent fatalities from a disease that is 100% vaccine-preventable albeit emphasises the need for comprehensive public health strategies, including vaccination campaigns, awareness programs improved healthcare infrastructure, communication between the human medical and veterinary sectors, and responsible ownership of dogs to mitigate the impact of rabies in such areas.

## Consent

The respondents provided informed oral consent to the investigators.

## Funding

DBT/Wellcome Trust India Alliance Intermediate Fellowship grant to Harish Kumar Tiwari (Grant number: IA/CPH/21/1/505597). The funding source had no involvement in the study design, data collection, interpretation, writing of the manuscript, or decision to submit the article for publication.

## CRediT authorship contribution statement

**Parimala Mohanty:** Conceptualization, Data curation, Writing – original draft. **Prasanta Kumar Boro:** Resources, Writing – original draft. **Samira Heydtmann:** Writing – review & editing. **Salome Durr:** Writing – review & editing. **Harish Kumar Tiwari:** Conceptualization, Funding acquisition, Writing – original draft, Writing – review & editing.

## Declaration of competing interest

The authors declare that there are no conflicts of interest regarding the publication of this article.

## Data Availability

Data will be made available on request.
